# Trends in Sexual Health of Gay, Bisexual, and Other Men Who Have Sex with Men, and Transgender Individuals: Apps Driven Testing Program for HIV and Other STIs in Barcelona, Spain (2016–2023)

**DOI:** 10.1007/s10900-023-01310-9

**Published:** 2023-12-08

**Authors:** Miguel Alarcón Gutiérrez, David Palma Díaz, Maria Lluïsa Forns Cantón, Laura Fernández-López, Patricia García de Olalla, Cristina Rius Gibert

**Affiliations:** 1 Department of Paediatrics, Obstetrics and Gynaecology and Preventive Medicine, Universitat Autònoma de Barcelona, Barcelona, Spain; 2 Epidemiology Service, Agència de Salut Pública de Barcelona, Barcelona, Spain; 3 Centre of Epidemiological Studies of HIV/AIDS and STI of Catalonia (CEEISCAT), Health Department, Generalitat de Catalunya, Badalona, Spain; 4 Department of International Health, Care and Public Health Research Institute—CAPHRI, Faculty of Health, Medicine and Life Sciences, Maastricht University, Maastricht, The Netherlands; 5 Centro de Investigación Biomédica en Red de Epidemiología y Salud Pública (CIBERESP), Madrid, Spain; 6 Institut de Recerca de l’Hospital de la Santa Creu i Sant Pau, Institut d’Investigació Biomédica Sant Pau, Barcelona, Spain

**Keywords:** Men who have sex with men, Gender minorities, HIV testing, STI testing, m-Health promotion, Epidemiology

## Abstract

Gay, bisexual and other men who have sex with men (GBMSM) and transgender individuals face heightened risks of HIV and other sexually transmitted infections (STIs). Surveillance within these populations is critical, and community testing services play a pivotal role in preventing and controlling HIV and STIs. This study investigates the trends in HIV, syphilis and hepatitis C (HCV) infections among participants in an apps-driven rapid test program from 2016 to 2023 in Barcelona, Spain, examining associated factors. Trend analysis utilized Wilcoxon-type test and associated factors were determined through multivariate logistic analysis. The prevalence of new HIV diagnosis was 1.81% (CI 1.18–2.64), active syphilis was 3.37% (CI 2.46–4.50) and acute HCV was 0.40% (CI 0.11–1.02). While infection rates showed no significant changes, there was significant increasing in sex work and chemsex and decreasing in condom use. Additionally, a peak in dating apps use for sex and a specific reduction in number of sexual partners were observed in 2020. Factors associated with HIV diagnoses included migrant status (aOR = 11.19; CI 2.58–48.53) and inconsistent condom use during the previous 12 months (aOR = 3.12; CI 1.02–9.51). For syphilis, associated factors were migrant status (aOR = 2.46; CI 1.14–5.29), inconsistent condom use (aOR = 3.38; CI 1.37–8.36), and chemsex practice during the previous 12 months (aOR = 2.80; CI 1.24–6.30). Our findings emphasize the need for tailored interventions, including culturally sensitive outreach for migrants and comprehensive strategies addressing substance use in sexual contexts. Technological innovations and targeted educational initiatives could reduce the burden of HIV and STIs within the GBMSM and transgender communities, providing valuable insights for public health strategies.

## Introduction

Gay, bisexual and other men who have sex with men (GBMSM), as well as transgender people, continue to face a disproportionate burden of HIV and other sexually transmitted infections (STIs). For example, they represented 40% of new HIV infections in Europe in 2021 and 68% of syphilis cases in 2019 [1, 2]. Multiple factors contribute to this disparity, including social stigma, discrimination and limited access to healthcare resources, leading to risky behaviours that increase the risk of HIV and STIs acquisition [3, 4]. Effective prevention and intervention strategies tailored to the unique challenges faced by these populations are urgently needed [5].

Surveillance within key populations, such as GBMSM and transgender individuals, plays a crucial role in improving their sexual health. It provides direct insights into infections and sexual behaviour, enabling timely responses. For instance, trend analysis of annual infection prevalence and specific variables can enhance surveillance, facilitating potential interventions in these key populations [6].

Community testing services are pivotal in preventing and controlling HIV and STIs as they can reach at-risk populations. Their significance is evident in behaviour change, increased testing frequency, viral suppression and early diagnosis of HIV and STIs [7–9]. International organizations recognize the importance of community-based testing for key populations, such as GBMSM, transgender individuals, migrants and sex workers [5, 10]. These services are commonly provided by community-led or peer-led organizations, often using point-of-care testing (POCT) strategies to reduce testing barriers [11].

Surveillance systems for HIV and STIs must be adaptable to specific settings and available resources. The incorporation of community testing significantly enhances the effectiveness of these systems by providing crucial and timely information [12, 13].

In recent years, dating apps and emerging technologies have gained attention as potential platforms for reaching GBMSM and transgender individuals and implementing preventive measures, complementing community efforts [14]. Their widespread use presents an opportunity to engage with these populations in innovative ways, such as promoting HIV/STI testing and providing access to essential healthcare services. Utilising these platforms, public health initiatives can directly target at-risk individuals, increase testing rates and facilitate the implementation of timely preventive strategies [5, 15, 16].

Dating apps have become essential tools for social and sexual interactions among GBMSM [17, 18]. They offer a convenient platform for individuals to connect with potential partners, leading to both personal and sexual encounters [19]. However, the use of dating apps in this population has been associated with an increased risk of acquiring HIV and other STIs, as well as engaging in higher-risk sexual behaviours, such as lower condom use and more sexual partners [20–22]. Furthermore, these risks extend beyond sexual behaviours, to include factors such as chemsex (the sexualized use of drugs), mental health problems and problematic use of the apps [23–26]. These factors contribute to the vulnerability of these populations to STIs, especially among those who are already HIV positive [27, 28].

The Public Health Agency of Barcelona (ASPB) has developed various interventions aimed to address the needs of GBMSM and transgender populations, such as Hepatitis A vaccination in response to outbreaks and the introduction of HIV testing in saunas in 2007 [29–31]. In 2015, ASPB initiated a pilot program to assess the response, acceptability and effectiveness of a POCT service for HIV and STIs testing and hepatitis A/B vaccination through dating apps [32]. This intervention expanded to community and primary health care centres between 2016 and 2019, with evaluations conducted to assess territorial differences [33]. Since 2019, the work has been centralized within the ASPB, establishing it as a “Rapid test program” for GBMSM and gender minorities. It offers regular testing services for HIV, syphilis, and hepatitis C (HCV), with the addition of papilloma virus vaccination for individuals under 27 years old. The intervention through dating apps and other technologies has intensified, with ASPB having eight active profiles on various dating apps, a WhatsApp Business account with a catalogue and automatic responses, and a website for scheduling testing appointments. Since 2021, the project also has an Instagram account, increasing participants recruited outside of dating apps.

Understanding the factors contributing to the transmission of HIV and other STIs among GBMSM, along with their temporal evolution, is crucial for developing effective prevention strategies [34]. By identifying associated factors, public health interventions can be tailored to address the specific needs of this population, reducing transmission rates and improving overall health outcomes [33, 35, 36]. This study aims to assess the annual trends in new diagnoses for HIV, acute syphilis and active HCV infection rates among GBMSM participating in ASPB’s Rapid Test Program over 8 years. Additionally, it aims to identify sociodemographic and behavioural variables associated with an increased risk for HIV, syphilis and hepatitis C infections.

## Methods

This was a study conducted in the city of Barcelona, Spain. The study subjects included were GBMSM and transgender individuals who voluntarily participated in any of the actions carried out by the researchers of the rapid testing program of the ASPB between the years 2016 and 2023 [32].

From 2016 to 2019, the recruitment was initiated by the provider through the ASPB profile in the dating apps, with invitations in the form of images, sent directly to dating apps users who were close to any of the diverse spots across the city. Since 2019, the recruitment has been voluntary initiated by the users themselves, allowing them to start a conversation on any available platform or request an appointment through the website.

Participants were offered HIV (Determine™ HIV Early Detect) and syphilis (Determine™ Syphilis TP) rapid testing and in some cases, HCV rapid testing (Oraquick® HCV). Individuals were tested only for not known previous infection for HIV, syphilis and/or HCV. Hepatitis A and B vaccination was offered in those non-vaccinated. Since 2019, HPV vaccination service was introduced through advertisements on some apps, offering free vaccination to GBMSM under 27 years old, as the protocol indicates.

All tested individuals received pre- and post-test counselling during the same visit. Reactive cases were referred for confirmation to an STI specific centre, their primary care centre, or their respective hospital, following the local protocol. Access to healthcare was provided to migrants without sanitary card for any STI diagnose. Although some patients continued visiting the centre to complete vaccination or regular testing, for study purposes, we included only the first visit for each participant.

Three different questionnaires have been used during this period, each related to different grants and research objectives. Nevertheless, sociodemographic information, sexual health and some sexual behaviours have remained consistent across the surveys. Those aspects that required harmonization were categorized as either lifetime or last year, depending on the availability of data.

### Variables

The dependent variables in this study were new HIV, acute syphilis and active HCV diagnoses. Independent sociodemographic variables included age as a numerical value and migrant status. Sexual health variables included any STI diagnosis the last 12 months, previous HIV testing and HIV serostatus. Behavioural variables included involvement in sex work, defined as receiving money or drugs in exchange for sex; the use of dating apps for sexual encounters; condom use during the last 12 months (always vs. not always), defining inconsistent condom use as the “not always” answer; the number of sexual partners during the last 12 months (less than 10, 10 or more); and the practice of chemsex during the last 12 months. Chemsex practice was defined as the use of methamphetamine, GHB/GBL or mephedrone with the intention of enhancing sexual experiences.

### Data Analysis

This was a cross-sectional and trends analysis. For trend analysis, we calculated both the total and annual prevalence proportions of new HIV, acute syphilis, and active HCV infections. The same methodology was applied to the main sociodemographic and behavioural variables, including migration status, involvement in sex work, use of dating apps for sexual purposes, condom use, number of sexual partners, and chemsex practice. Additional trend analysis was conducted for prophylaxis pre-exposition (PrEP) usage, although this data was only available from 2021 onwards. Trend tests were performed using a Wilcoxon-type test for each variable, and graphical content was generated by including trend test p-values.

Descriptive univariate analysis was performed on the sociodemographic, sexual health and behavioural characteristics of the tested individuals, presenting proportions for categorical data and medians with interquartile range (IQR) for numerical variables. In addition, bivariate analysis was conducted to examine the relationship with the main outcome using the chi-square test, presenting proportions and p-values. Analysis for associated factors to active HCV infections were not carried out due to the small sample size of cases. Lastly, a multivariate logistic regression analysis was performed to identify factors associated with HIV/STI infections, presenting the adjusted Odds Ratios (aOR), 95% confidence intervals (CI) and p-values. For model construction, we explored all potential equations [37], adjusting for variables with > 90% confidence, age and dating apps using for sex, given its epidemiological significance.

### Ethical Aspects

The study was approved by the research ethics committee of Parc de Salut Mar (No. 2016/6984/I). All data were anonymized, and informed consent was obtained. Information was handled in accordance with the data storage procedures stipulated in Organic Law 3/2018 on personal data protection and guarantee of digital rights.

## Results

**Fig. 1:**
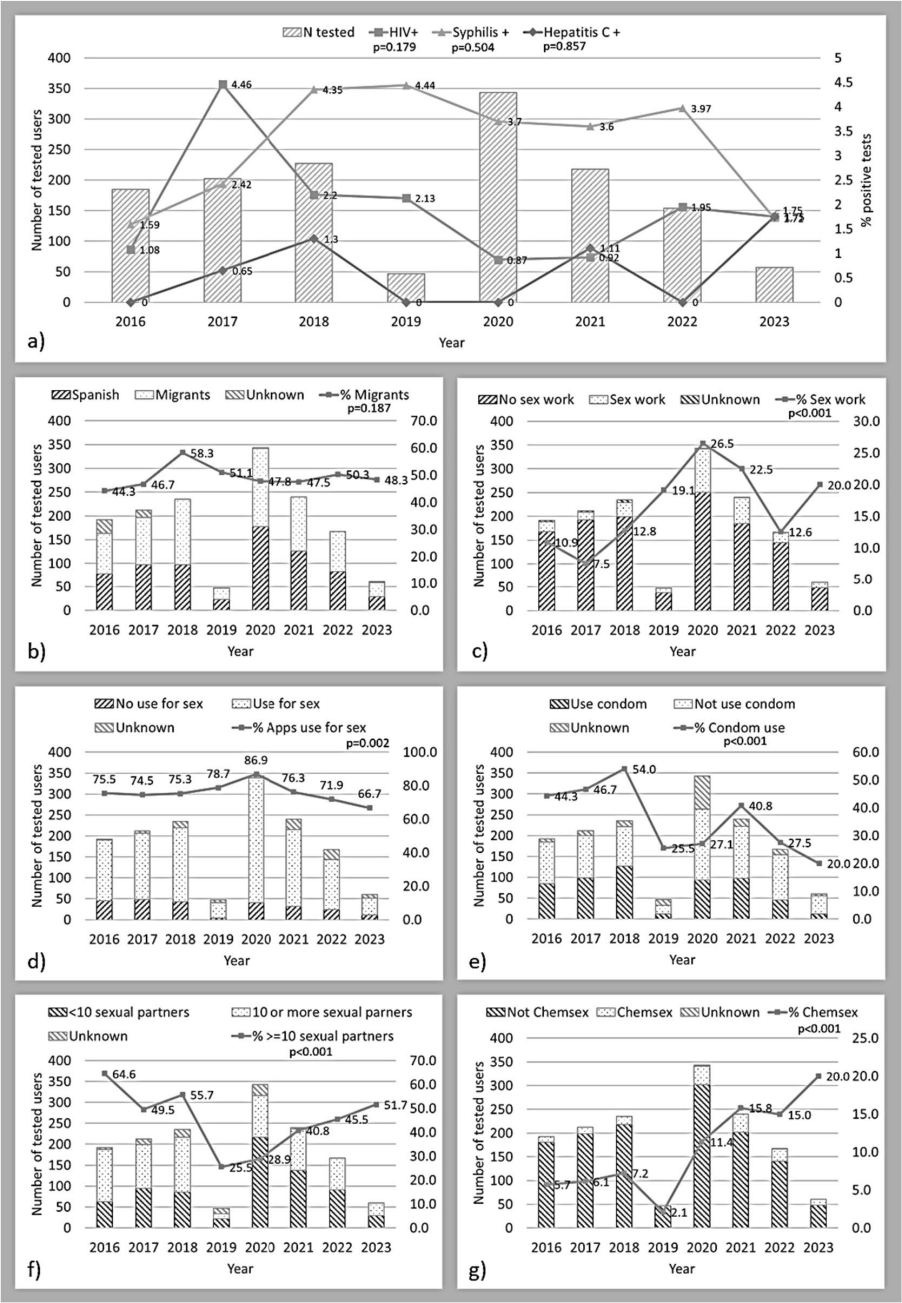
Annual trends of HIV, syphilis and hepatitis C infection, demographic and behavioural variables in a rapid testing through dating apps for GBMSM in Barcelona, Spain. 2016–2023. P-values of test for trend across ordered groups. **a** HIV syphilis and hepatitis C proportion and number of total tested people per year; **b** Proportion of migrant people; **c** Proportion of people who reported having sex for money or goods; **d** Proportion of people who use dating apps for sex meetings; **e** Proportion of people reporting not always condom use during last 12 months; **f** Proportion of people reporting 10 or more sexual partners during last 12 months; **g** Proportion of people who reported chemsex practice (use of methamphetamine, GHB/GBL or mephedrone for sex)

In total, 1502 tested GBMSM and transgender were included. The prevalence of new diagnoses for HIV was 1.81% (CI 1.18–2.64), 3.37% (CI 2.46–4.50) for active syphilis and 0.40% (CI 0.11–1.02) for acute HCV. Annual trends can be observed in Fig. 1. Based on the testing data, the total number of tested individuals was highest in 2020 and lowest in 2019. There were not statistically significant changes in the annual trends for HIV, syphilis or HCV infections.

According to the independent variables (Fig. 1), the proportion of migrant people showed stable trends around 50% (p = 0.187). Sex work had a significant increase across the years, rising from 10.9% in 2016 to 20.0% in 2023, with a peak of 23.7% in 2021 (p < 0.001). The use of dating apps for sexual purposes remained relatively stable at around 75%, but experienced a significant peak in 2020, reaching 86.9% (p = 0.002). Consistent use of condom decreased significantly from 44.3% in 2016 to 20.0% in 2023 (p < 0.001). The use of PrEP (not shown) did not exhibit significant changes (p = 0.234), with a proportion of 11.9% from 2021 to 2023. The number of sexual partners showed a significant decrease, with a prevalence of 64.6% of individuals having 10 or more partners in 2016 to 51.7% in 2021, with the lowest prevalence in 2019 and 2020 (p < 0.001). The practice of Chemsex experienced a substantial and sustained increase, rising from 6% in 2016 to 20.0% in 2023 (p < 0.001).

Univariate descriptive analysis is presented in Table 1. The median age of the tested users was 33 years old (IQR 26–38). Among them, 49.1% were foreign nationals, of which 69.4% were from Latin America and the Caribbean region of the World Health Organization, 9.1% from Southern Europe and 5.0% from Eastern Europe. According to health variables, 6.9% were new testers, 2.6% were people with known diagnosis of HIV and 23.0% had an STI in the previous 12 months. Behavioural variables showed that 17.0% had engaged in sex work, 77.4% had used dating apps for sexual encounters, 38.4% had consistently used condoms in the past 12 months, 45.2% had 10 or more sexual partners during the last 12 months and 10.4% had engaged in chemsex.

**Table 1:** Tested GBMSM of a preventive program through dating apps

Variables	Total sample (n = 1502)		HIV tested (n = 1413)					Syphilis tested (n = 1258)				
			Negative		Positive		p-value^a^	Negative		Positive		p-value^a^
	N	%	N	%	N	%		N	%	N	%	
Age (Median[IQR])	33	[26–38]	33	[26–38]	32	[26–33]	0.4801^b^	33	[26–39]	33	[25–37]	0.9473^b^
Migration situation												
No	717	47.7	686	48.5	2	7.7	< 0.001	616	48.9	15	34.1	0.063
Yes	738	49.1	681	48.2	24	92.3		609	48.3	27	61.4	
Unknown	47	3.1	46	3.3	0	0.0		35	2.8	2	4.5	
Ever tested for HIV												
No	104	6.9	100	7.1	2	7.7	0.960	89	7.1	4	9.1	0.462
Yes	976	65.0	935	66.2	18	69.2		799	63.4	24	54.5	
Unknown	422	28.1	378	26.8	6	23.1		372	29.5	16	36.4	
Living with HIV												
No	1360	90.5						1148	91.1	39	88.6	0.197
Yes	39	2.6						23	1.8	2	4.5	
Unknown	103	6.9						89	7.1	3	6.8	
Previous STI*												
No	1145	76.2	1090	77.1	20	76.9	0.920	983	78.0	34	77.3	0.946
Yes	346	23.0	312	22.1	6	23.1		267	21.2	9	20.5	
Unknown	11	0.7	11	0.8	0	0.0		10	0.8	1	2.3	
Sexual work												
No	1232	82.0	1164	82.4	19	73.1	0.346	1034	82.1	31	70.5	0.068
Yes	255	17.0	236	16.7	6	23.1		214	17.0	12	27.3	
Unknown	15	1.0	13	0.9	1	3.8		12	1.0	1	2.3	
Dating apps use for sex												
No	249	16.6	232	16.4	6	23.1	0.336	196	15.6	10	22.7	0.161
Yes	1163	77.4	1097	77.6	18	69.2		987	78.3	30	68.2	
Unknown	90	6.0	84	5.9	2	7.7		77	6.1	4	9.1	
Inconsistent condom use*												
No	577	38.4	557	39.4	4	15.4	0.010	493	39.1	7	15.9	0.002
Yes	764	50.9	705	49.9	19	73.1		626	49.7	31	70.5	
Unknown	161	10.7	151	10.7	3	11.5		141	11.2	6	13.6	
Number of sexual partners*												
< 10	743	49.5	712	50.4	8	30.8	0.052	658	52.2	14	31.8	0.005
10 or more	679	45.2	624	44.2	16	61.5		532	42.2	28	63.6	
	80	5.3	77	5.4	2	7.7		70	5.6	2	4.5	
Chemsex practice*												
No	1343	89.4	1280	90.6	22	84.6	0.284	1141	90.6	33	75.0	0.003
Yes	156	10.4	130	9.2	4	15.4		118	9.4	10	22.7	
Unknown	3	0.2	3	0.2	0	0.0		1	0.1	1	2.3	
Actual HIV test												
Negative	1413	98.2										
Positive	26	1.8										
Actual syphilis test												
Negative	1260	96.6										
Positive	44	3.4										
Actual HCV test												
Negative	996	99.6										
Positive	4	0.4										

Univariate and bivariate descriptive analysis. Barcelona, Spain, 2016–2023

*During last 12 months

^a^Chi-square test, p-values do not include missing values

^b^U Mann–Whitney

Bivariate analysis (Table 1) showed that variables associated with new HIV diagnoses in the sample were a higher migrant situation (p < 0.001) and lower condom usage (p = 0.010). A marginally associated variable was a higher number of sexual partners (p = 0.052). In the case of acute syphilis diagnosis, associated variables were lower condom use (p = 0.006), a higher number of sexual partners (p = 0.005), and a higher proportion of chemsex practice (p < 0.001).

Finally, in the multivariate analysis (Table 2), the associated factors with new HIV diagnosis were migrant status (aOR = 11.19; CI 2.58–48.53), and inconsistent condom use during the previous 12 months (aOR = 3.12; CI 1.02–9.51). The associated factors with acute syphilis diagnosis were migrant status (aOR = 2.46; CI 1.14–5.29), inconsistent condom use during the previous 12 months (aOR = 3.38; CI 1.37–8.36), and chemsex practice during the previous 12 months (aOR = 2.80; CI 1.24–6.30).

**Table 2:** Tested GBMSM of a preventive program through dating apps

	HIV diagnosis			Syphilis diagnosis		
	aOR	CI95%	p-value	aOR	CI95%	p-value
Age	1.00	(0.96–1.05)	0.917	1.00	(0.96–1.03)	0.819
Migration situation						
No	1 (reference)			1 (reference)		
Yes	11.19	(2.58–48.53)	0.001	2.46	(1.14–5.29)	0.021
Dating apps use for sex						
No	1 (reference)			1 (reference)		
Yes	0.47	(0.18–1.24)	0.126	0.56	(0.24–1.31)	0.181
Inconsistent condom use*						
No	1 (reference)			1 (reference)		
Yes	3.12	(1.02–9.51)	0.046	3.38	(1.37–8.36)	0.008
Number of sexual partners*						
< 10	1 (reference)					
10 or more	1.10	(1.00–1.21)	0.058			
Chemsex practice*						
No				1 (reference)		
Yes				2.80	(1.24–6.30)	0.013

Multivariate logistic analysis of factors associated to HIV and syphilis diagnosis. Barcelona, Spain, 2016–2023

*aOR* adjusted odds ratio, *CI95%* confidence interval for 95%

*During last 12 months

## Discussion

### Trends Analysis

The study’s results provide a comprehensive overview of the annual prevalence of new HIV, acute syphilis and active HCV infections among the tested population. Our study reveals a higher prevalence compared to the annual incidence in men within the city, with a 0.02% of HIV infection and 0.14% of syphilis in 2022 [38]. In contrast, the EMIS-2017 study reported a prevalence of 1.3% for HIV and 5.4% for syphilis among Spanish GBMSM^39^, which closely mirrors our findings.

The lack of significant changes in the trend of new HIV and acute syphilis diagnoses over the 8 year period, as it has been reported in the city rates [39], highlight the persistent challenges in prevention and the need for innovative interventions. Unlike European data, active HCV infections remained stable in our study [40]. This may be because HCV infections primarily affects to HIV-positive GBMSM, who were less likely to visit our facilities due to ongoing care. A recent study in Spain indicated a 0.52% prevalence among HIV-negative GBMSM, slightly higher than our findings, and identified associated factors such as the use of sexualized drugs associated with infection [41].

Notably, the number of individuals engaging with the ASPB’s Rapid Test Programme for the first time increased in 2020, during the COVID-19 pandemic. This may be attributed to increased apps usage during the COVID-19 pandemic [42] and the closure of other sexual health services, driving more consultations with ASPB’s service [43]. This increasing may, also, explain the significant peak in the use of dating apps for sexual encounters in 2020.

Analysing the trends for independent variables further enriches the study’s findings and provides an opportunity to integrate this approach into public health surveillance to enhance interventions. As previously described, integrating community-based testing data into surveillance systems could improve monitoring and provide epidemiological insights about key populations [44].

The substantial proportion of migrant individuals suggests that dating apps are an effective platform for reaching this at-risk group. Existing literature indicates that migrant individuals face limited access to health promotion programmes and a lower proportion of linkage-to-care due to factors like discrimination and violence [45]. This is particularly significant when we consider that the majority of migrants come from developing countries. Additionally, a syndemic effect of being a migrant with HIV/ST-related risk behaviours and substance use further emphasizes the need for tailored interventions [46].

The significant increase in sex work, likely facilitated by dating apps [47], is noteworthy and may be influenced by economic factors. Moreover, men’s sex work has been associated with a high prevalence of HIV and STIs, risky behaviours as substance use and low condom usage, especially among migrant sex workers [48–50].

The decline in consistent condom use raises concerns about safe sexual practices, aligning with previous research indicating decreasing condom use among dating app users [20]. While PrEP, introduced in Spain in 2019, may contribute to this trend, it does not fully explain the low proportion of condom use. This highlights the need for renewed efforts to promote safer sex practices, including condom use, especially when engaging with unknown sexual partners through dating apps.

Changes in the number of sexual partners also warrant consideration. The lowest proportion was observed in 2019, possibly due to a small sample size. However, the decrease in 2020 was likely a result of COVID-19 restrictions, reducing social contact [42]. In 2021, this trend reversed rapidly, but from 2022 onwards, the increase has been less pronounced, possibly influenced by the global and local Monkeypox outbreak, where sexual contact among men was associated with its spread [51].

One of the most striking findings is the significant increase in chemsex practice over the study period. The association of Chemsex with higher infection rates highlights the interconnectedness of substance use and sexual health within this population and, more broadly among dating apps users [27]. Addressing chemsex requires comprehensive strategies targeting substance abuse and sexual risk behaviours, with dating apps serving as a potential platform for such initiatives [52].

### Factors Associated to HIV and Syphilis Infection

The multivariate analysis delves deeper into the factors associated with new HIV and acute syphilis diagnoses. Migrant status emerges as a prominent risk factor for both infections, underscoring the need for culturally sensitive outreach and support for migrant individuals within the GBMSM community. Specific studies on migrants who use dating apps should be conducted, considering common factors associated with their health and well-being [45].

Inconsistent condom use appears as a common risk factor, emphasizing the need for educational campaigns promoting safer sex practices, including alternatives such as PrEP. Additionally, a more comprehensive evaluation of the concept of safe sex is warranted, beyond the sole assessment of condom use.

Furthermore, the association between chemsex practice and acute syphilis diagnosis aligns with previous research demonstrating the link between substance use and STI acquisition [53]. This underscores the urgent need for interventions addressing substance use and its impact on sexual behaviours.

### Limitations and Strengths

The study’s limitations should be considered in interpreting the results and planning future research. The cross-sectional design does not establish causality but provides valuable information on associations and annual trends. The geographic focus on Barcelona may limit external validity, but similar behavioural and epidemiological trends in European big cities enhance the study’s relevance [54]. Although is collected since 2021, the absence of gender identity data in the prior questionnaire is a limitation for evaluating this very important variable, necessitating changes in data collection. Harmonization of variables strengths surveillance systems, so it should be considered when developing new instruments.

Despite these limitations, the study’s strengths lie in the interventions conducted by the Public Health Agency and their epidemiological surveillance department, developing a service that goes beyond outbreak response. The implemented measures lead us to believe that these interventions are necessary, as well as being a part of the routine duties of healthcare surveillance workers. Barcelona’s unique characteristics as a destination for GBMSM migration and tourism have contributed to the rapid spread of STIs. The results encourage the continued development of better interventions for all sexually active populations, with a real-time focus on key populations. The program has also established an improved surveillance method, offering timely information on sexual behaviours among GBMSM and transgender populations in the city, which can inform future interventions.

## Conclusion

The present work shows a high prevalence of new HIV and acute syphilis diagnosis among GBHSH and transgender individuals who participate in a rapid test program through social networks. Also, there were an increasing trend in sex work, chemsex and decreasing in condom use, with a peak in apps use for sex in 2020. This study provides crucial insights into the evolving landscape of HIV, syphilis and HCV infection and factors associated. The findings shed light on persistent challenges, such as low condom use, high-risk sexual behaviours, and the growing concern of chemsex. Addressing these challenges requires multifaceted interventions that combine technological innovations with targeted education and support. As well, this program has improved surveillance data, which can be used for future interventions in the city. By understanding the complex interplay of sociodemographic and behavioural factors, public health initiatives can tailor interventions to address the unique needs of the GBMSM community and work towards reducing the burden of HIV and STIs.
